# The future of bioorthogonal-chemistry for targeting of exosomes in precision medicine

**DOI:** 10.18632/oncotarget.28323

**Published:** 2022-12-06

**Authors:** Mujib Ullah

**Keywords:** precision medicine, bioorthogonal, exosomes

**Figure 1:**
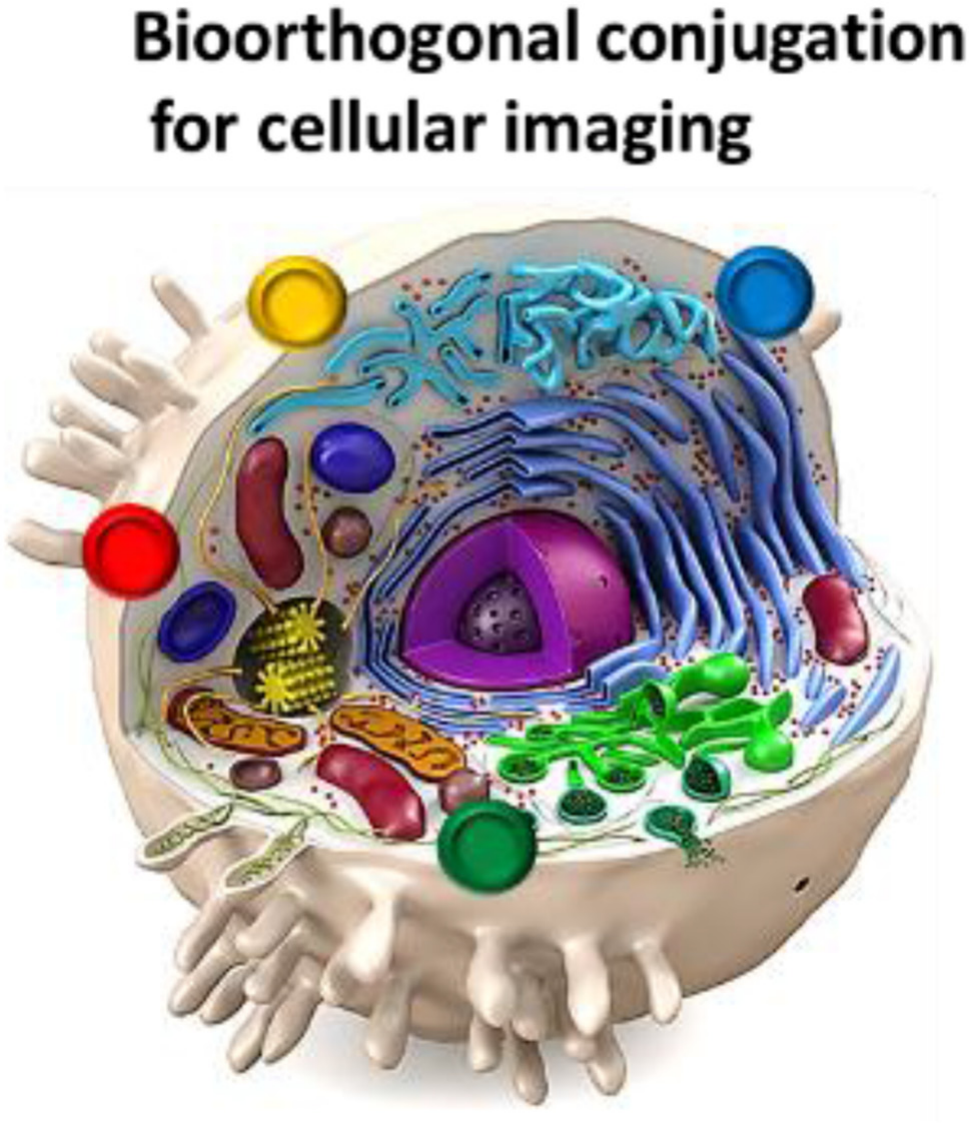
Schematic illustration showing tracking of exosomes labeled by phospholipid-based bioorthogonal conjugation. The term bioorthogonal chemistry refers to any chemical reaction that can occur in the presence of other rich chemical functionalities found in biological systems without interacting or interfering with native biochemical processes. In the cell, bioorthogonally – activated components react specifically, selectivity and bioorthogonally-conjugated exosomes can be used for cellular imaging.

The combination of chemistry and biology has accelerated the field of next generation of precision medicine. Bioorthogonal is the name of a chemical reaction that can occur inside of living cells without interfering the naïve biological process [1, 2]. This new approach allows scientists to explore cells and track biological processes without disrupting the normal biology of the cells [2]. The applications of this innovative field is ranging from bioengineering, to real time tracking of the drugs [1]. The bioorthogonal approach can be used in precision medicine for precise imaging. Given the incredible precision this approach can be used for the real time tracking of extracellular vesicles or exosomes (Figure 1).

The concept of bioorthogonal chemistry has inspired a generation of biologists to think about RNA editing and bioengineering of exosomes [3, 4]. For instance, current loading techniques in exosome vesicles, such as electroporation, heat shock, sonication, and ultracentrifugation may destroy the membrane proteins and hamper the targeting ability of exosome vesicles [4, 5]. However, controlled, precise and targeted biorthogonal reaction may overcome this challenge. The metabolic cargo of exosomes can be labelled with biorthogonal conjugations [6]. In one study the biotin-clicked exosomes encapsulated into exosomes and used for targeting the recipient cells [7–9]. In another study, Desmoglein-1c is enriched in exosomes linked by bioorthogonal system effectively homed to myocardial tissues [9].

The fluorescence labeling of Cy5-Exo was noninvasively tracked and imaged in tumor-bearing mice [10, 11]. Stem cell derived exosomes, allowing localized prodrug activation [11]. To showcase the potential of bioorthogonal reaction in clinical biomedical applications the following question should be investigated further. Can bioorthogonal chemistry help in the development of more powerful bioimaging and biosensing techniques? Can the combination of exosomes with biorthogonal chemistry overcome some of the current translational hurdles in precision medicine? Can new drugs be designed inside humans? Can the exosomes cargo be detected by conjugation chemistry? Can we track the metabolites encapsulated inside the exosomes? Can pharmaceuticals be synthesized inside living system? How many orthogonal reactions can be performed in one time? What is the half life of these reactions? Biorthogonal reactions are rapid but what is the speed of reactions? More investigation is needed to explore this new avenue of science.
